# Dexmedetomidine Protects against Myocardial Ischemia/Reperfusion Injury by Ameliorating Oxidative Stress and Cell Apoptosis through the Trx1-Dependent Akt Pathway

**DOI:** 10.1155/2020/8979270

**Published:** 2020-11-25

**Authors:** Zhi-lin Wu, Jacques Robert Jeppe Davis, Yi Zhu

**Affiliations:** ^1^Institute of Anesthesia and Critical Care Medicine, Department of Anesthesiology, Union Hospital, Tongji Medical College, Huazhong University of Science and Technology, Wuhan, China; ^2^Department of Paediatric Orthopaedics Royal Children's Hospital, Glasgow, Scotland, UK

## Abstract

Dexmedetomidine (Dex) was reported to reduce oxidative stress and protect against myocardial Ischemia/Reperfusion (I/R) injury. However, the molecular mechanism involved in its antioxidant property is not fully elucidated. The present study was aimed at investigating whether the Trx1/Akt pathway participated in the cardioprotective effect of Dex. In the present study, I/R-induced myocardial injury in isolated rat hearts and OGD/R-induced injury in H9c2 cardiomyocytes were established. Our findings suggested that Dex ameliorated myocardial I/R injury by improving cardiac function, reducing myocardial apoptosis and oxidative stress, which was manifested by increased GSH and SOD contents, decreased ROS level, and MDA generation in both the isolated rat hearts and OGD/R-treated H9C2 cells. More importantly, it was found that the level of Trx1 was preserved, and Akt phosphorylation was significantly upregulated by Dex treatment. However, these effects of Dex were abolished by PX-12 (a specific Trx1 inhibitor) administration. Taken together, this study suggests that Dex plays a protective role in myocardial I/R injury, improves cardiac function, and relieves oxidative stress and cell apoptosis. Furthermore, our results present a novel signaling mechanism that the cardioprotective effect of Dex is at least partly achieved through the Trx1-dependent Akt pathway.

## 1. Introduction

Ischemic heart disease is the leading cause of death in the whole world [1]. Although timely reperfusion is the most effective therapeutic strategy, reperfusion itself can result in lethal cardiac damage. This process is known as myocardial Ischemia/Reperfusion (I/R) injury [2]. An imbalance between oxidative stress and antioxidant mechanisms is observed in myocardial I/R, which is associated with the excessive generation of reactive oxygen species (ROS) activated by reperfusion [3], eventually leading to cell death and apoptosis in the heart [4]. Recent studies have shown that targeted antioxidants could effectively ameliorate oxidative stress and enhance the efficacy of treatments for I/R injury. Therapeutic strategies against oxidative stress in myocardial I/R injury have become more promising in recent years.

Dexmedetomidine (Dex), a highly selective alpha 2 adrenergic receptor agonist, is widely used for ICU sedation and auxiliary anesthesia. Interestingly, it has been found that this sedative exerts a capability of protection directly against I/R injury in multiple organs. Recent studies showed that Dex could inhibit the release of proinflammatory cytokines and attenuate I/R-related apoptosis in acute kidney injury, cerebral ischemia injury, and hepatic I/R injury, which might be ascribed to reducing the production of ROS [5–7].

The myocardium has endogenous reducing mechanisms that are involved in the cell's counteraction against this oxidative damage. Of them, thioredoxin 1 (Trx1) is a critical molecule to protect the heart against I/R injury and ROS damage [8, 9]. Using Trx1 transgenic mice, Adluri et al. showed that Trx1 overexpression conferred a protective effect against I/R injury and decreased the infarct size by protein kinase B (Akt) activation, which is a crucial regulator of metabolism and cell survival [10]. Moreover, several studies have shown that Akt activation was enhanced with the administration of Dex in myocardial I/R models [11, 12]. However, whether Trx1 is involved in the reduction of ROS and Akt activation by Dex is not fully elucidated yet.

Based on the above observations, we hypothesized that Dex could reduce oxidative stress and cell apoptosis by regulation of the signaling pathway Trx1/Akt, thus protecting the heart against I/R injury. In the present study, we investigated the effect of Dex pretreatment on oxidative stress and cell apoptosis in myocardial I/R models. Furthermore, the underlying mechanism of the Trx1/Akt pathway involved in Dex against I/R injury was explored.

## 2. Material and Methods

### 2.1. Animals and Drugs

Adult male Sprague-Dawley rats (250–300 g) were purchased from the Hunan Weasley Scene of Experimental Animals Co., Ltd. (Hunan China). All animal protocols were performed following the U.S. National Institutes of Health guidelines (NIH Publication No. 85-23, revised 1996) and approved by the Animal Care and Use Committee of Tongji Medical College of Huazhong University of Science and Technology. Dex was purchased from Jiangsu Hengrui Pharmaceutical Co., Ltd. (Lianyungang, China), and 1-methyl propyl 2-imidazolyl disulfide (PX-12) was purchased from Cayman Chemical (Michigan, USA).

### 2.2. Isolated Heart Perfusion Experiment

A myocardial I/R model was established as previously described [13]. Briefly, rats were anesthetized with sodium pentobarbital (50 mg/kg, i.p.) and injected with heparin (500 IU, i.p.). Then, the hearts were excised and fixed on an apparatus and perfused with Krebs-Henseleit (K-H) buffer as previously described [13]. The rats were randomly divided into three groups (*n* = 8): control group—the hearts were perfused continuously for 120 min; I/R (I/R) group—the hearts were perfused for a 30 min stabilization and then underwent 30 min ischemia, followed by 60 min reperfusion; and I/R+Dex group—the hearts were perfused with 10 nM Dex during 30 min stabilization, and then, the rest of the procedures were the same as in the I/R group. During perfusion, hemodynamic parameters were recorded continuously via a water-filled balloon placed into the left ventricle and attached to a pressure transducer after the heart was mounted on a Langendorff apparatus (ADInstruments, Australia). The data were collected and analyzed using a PowerLab data acquisition system with LabChart 7 software (ADInstruments, Australia). Measured parameters included left ventricle developed pressure (LVDP), left ventricle end-diastolic pressure (LVEDP), and maximum LVDP increase (+dp/dt) and decrease (−dp/dt) rates. At the end of the perfusion, hearts were collected for the determination of the following experiments.

### 2.3. Cell Culture and the Induction of OGD/R

The H9C2 cardiac cell line was obtained from ATCC and cultured in high-glucose DMEM supplemented with 10% fetal bovine serum (FBS) and 1% penicillin-streptomycin in a humidified incubator (5% CO_2_, 95% air, 37°C). To mimic the I/R injury model on H9C2 cells, the oxygen-glucose deprivation/reperfusion (OGD/R) model was generated by bathing the cells in glucose-free DMEM within an anaerobic chamber (5% CO_2_, 95% N_2_) at 37°C for 12 h (oxygen-glucose deprivation (OGD)). Then, the cells were incubated with normal culture medium under normoxic conditions (95% air, 5% CO_2_) for 6 h at 37°C (reperfusion (R)). Normoxic control cells (control group) were treated identically without OGD/R. Furthermore, H9C2 cells were exposed to OGD/R with or without Dex preconditioning (1 *μ*M) for 1 h. The Trx1 inhibitor PX-12 (5 *μ*M) was pretreated for 30 min before OGD/R.

### 2.4. Western Blotting

Western blot analysis was performed as described previously [14]. Briefly, proteins were extracted and separated on 10-12% SDS-PAGE gels, and then, the gels were transferred to polyvinylidene difluoride (PVDF) membranes (Millipore, USA). The PVDF membranes were blocked in 5% nonfat milk dissolved in Tris-buffered saline with Tween 20 (TBST) at room temperature for 1 h. Thereafter, the PVDF membranes were incubated with the following primary antibodies: anti-phospho-Akt (Ser473), anti-Akt (1 : 1,000; Cell Signaling Technology, USA), and anti-Trx1 (1 : 1,000; ABclonal, China) at 4°C overnight, followed by incubation with the HRP-conjugated anti-rabbit secondary antibody (1 : 5,000; Proteintech, China). Target protein bands were visualized using the ChemiDocXRS+ chemiluminescence imaging system (Bio-Rad, USA) and quantified using Image Lab software (Bio-Rad, USA).

### 2.5. Determination of Myocardial Infarct Size and Cell Apoptosis

The hearts of each group were collected at the end of reperfusion and snap-frozen at -20°C for 2 h and then cut into 2 mm thick sections. Heart sections were incubated in a 1% solution of 2,3,5-triphenyltetrazoliumchloride (TTC; Ameresco, USA) for 15 min at 37°C. The sections were fixed in 4% paraformaldehyde-phosphate-buffered saline (PBS) solution at 4°C. Normal tissue was stained red while the infarcted tissue was white (unstained). Infarct size was scored using the ImageJ software (NIH, USA) and was expressed as a percentage of the total ventricular area.

Apoptosis was evaluated using the terminal dUTP nick-end labeling (TUNEL) staining kit (Roche, Switzerland) based on the manufacturer's instructions. Images were captured using a light microscope (Olympus, BX53), and the apoptotic index was represented as the ratio of the number of apoptotic nuclei to the total number of nuclei counted ×100%.

### 2.6. Biochemical Analysis

The hearts were collected and homogenized in RIPA lysis buffer. After centrifugation at 12,000 g for 10 min, the supernatant was collected. Then, contents of glutathione (GSH) and malondialdehyde (MDA), as well as activity of the antioxidative enzyme superoxide dismutase (SOD), were measured according to the instructions of the corresponding assay kit (Jiancheng Biotech, Nanjing, China). Besides, the process of analysis in H9c2 cells was similar to the method described above.

### 2.7. ROS Production Assay

Cellular ROS production was assayed using the ROS assay kit (Beyotime Biotech, China). Briefly, H9C2 cells were treated according to the conditions indicated, incubated with 10 *μ*M 2′,7′-dichlorofluorescein diacetate (DCFH-DA) in the dark for 20 min at 37°C, and washed three times with serum-free medium. DCFH-DA fluorescence was detected at 525 nm using flow cytometry.

### 2.8. Cell Viability Assessment

In an in vitro study, cell viability was evaluated using the 3-(4,5-dimethylthiazol-2-yl)-2,5-diphenyltetrazolium bromide (MTT) assay kit (Sigma, China) according to the manufacturer's instructions. Optical density (OD) was measured using a microplate reader (Thermo, USA) at 568 nm.

### 2.9. Statistical Analysis

All data were presented as the mean ± standard deviation (SD) value of at least three independent experiments. Statistical analyses were conducted using GraphPad Prism 7 (GraphPad Software Inc., USA). Data were analyzed by a one-way ANOVA (two-way ANOVA for the isolated heart perfusion study) followed by post hoc Tukey's test for multiple comparisons. *P* < 0.05 was considered statistically significant.

## 3. Results

### 3.1. Dex Improved Hemodynamic Effects of Hearts That Underwent I/R

**Figure 1:**
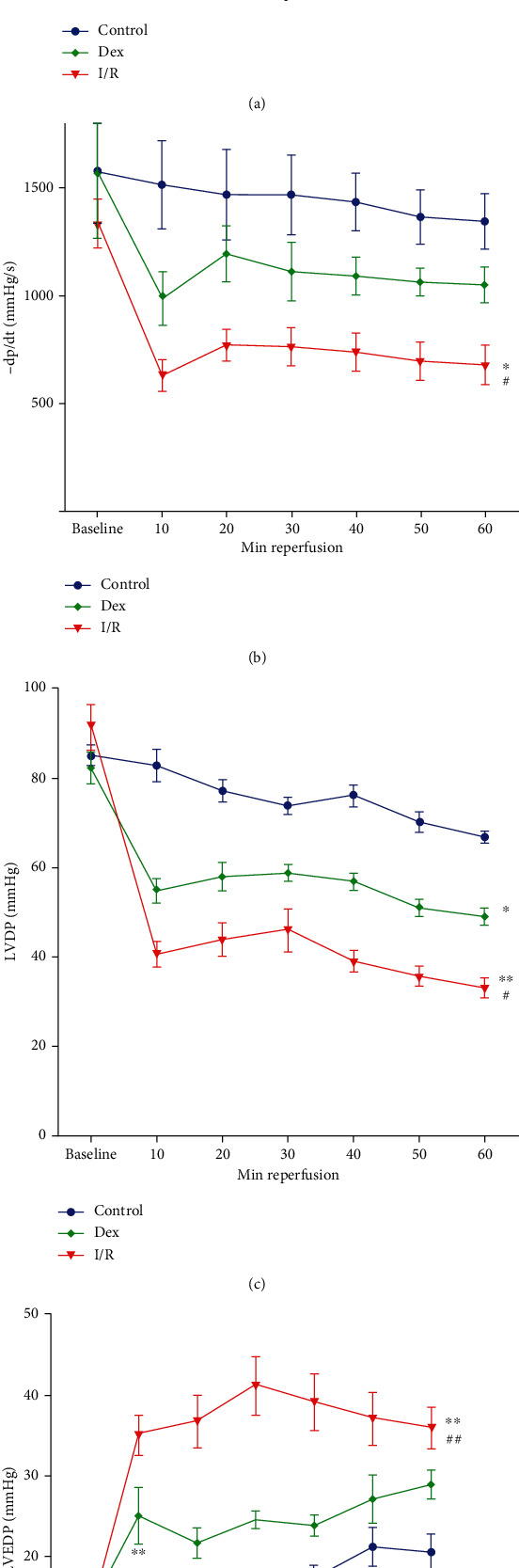
Dexmedetomidine (Dex) ameliorated cardiac function following I/R (I/R). The hearts were isolated and underwent 30 min ischemia, followed by 60 min reperfusion. Recovery of left ventricular functional assessments is shown: (a) +dp/dt, (b) -dp/dt, (c) LVDP, and (d) LVEDP. Baseline indicates preischemic values; the rest are minutes of reperfusion. Values are expressed as mean ± SD (*n* = 6/group). ^∗^*P* < 0.05, ^∗∗^*P* < 0.01 vs. control group. ^#^*P* < 0.05, ^##^*P* < 0.01 vs. I/R group.

The influence of Dex treatment on the recovery of cardiac function following I/R was investigated. As shown in Figure 1, during reperfusion, ischemic hearts demonstrated a worse performance in postischemic hemodynamic parameters (LVDP 39.76 ± 3.93 mmHg, +dp/dtmax 1261 ± 22.43 mmHg/s, −dp/dtmin 713 ± 53.41 mmHg/s, and LVEDP 54.71 ± 5.54 mmHg) as compared to hearts without ischemia (LVDP 74.36 ± 5.53 mmHg, +dp/dtmax 2316 ± 75.02 mmHg/s, −dp/dtmin 1432 ± 66.03 mmHg/s, and LVEDP 16.52 ± 3.9 mmHg) (*P* < 0.01, respectively; LVEDP *P* < 0.05). Meanwhile, Dex dramatically improved the recovery of LVDP (54.71 ± 3.93 mmHg), +dp/dtmax (1722 ± 26.23 mmHg/s), −dp/dtmin (1082 ± 68.57 mmHg/s), and LVEDP (25.24 ± 2.54 mmHg) compared with I/R alone (*P* < 0.05, respectively; LVEDP *P* < 0.01). At the end of reperfusion, there were no differences between groups Dex and control in these parameters.

### 3.2. Dex Protected the Heart from I/R Injury

**Figure 2:**
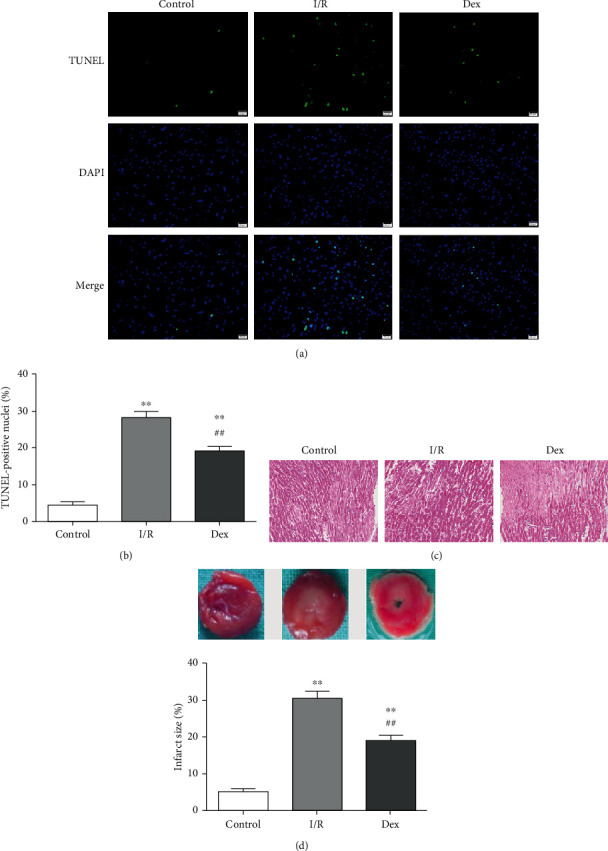
Myocardial infarct size and cardiomyocyte apoptosis in I/R rats. (a) Representative TUNEL staining images. Magnification (400x). (b) Statistical analysis of (a). (c) Representative photomicrographs of HE-stained left ventricular tissue sections. Original magnification (×200). (d) Myocardial infarct size. Data are shown as mean ± SD (*n* = 6/group). ^∗^*P* < 0.05, ^∗∗^*P* < 0.01 vs. control group. ^#^*P* < 0.05, ^##^*P* < 0.01 vs. I/R group.

To examine the effects of Dex on myocardial I/R injury in rats, myocardial tissue injury was detected by TTC staining and TUNEL assay. The results manifested that I/R injury significantly increased the apoptosis rate in myocardial cells from 4.36 ± 2.32% (control) to 28.24 ± 4.31% (I/R). Moreover, the infarct size percentage was increased to 30.37 ± 4.83% compared with 5.05 ± 2.06% in nonischemic controls (*P* < 0.01, respectively, Figures 2(b) and 2(d)). In contrast, the apoptosis rate of Dex-treated hearts (19.21 ± 3.08%) was significantly lower than that of the I/R group (*P* < 0.01, Figure 2(b)). Meanwhile, Dex also reduced the infarct size (18.96 ± 3.59%) compared with I/R alone (*P* < 0.01, Figure 2(d)).

### 3.3. Dex Ameliorated I/R-Induced Oxidative Stress

**Figure 3:**
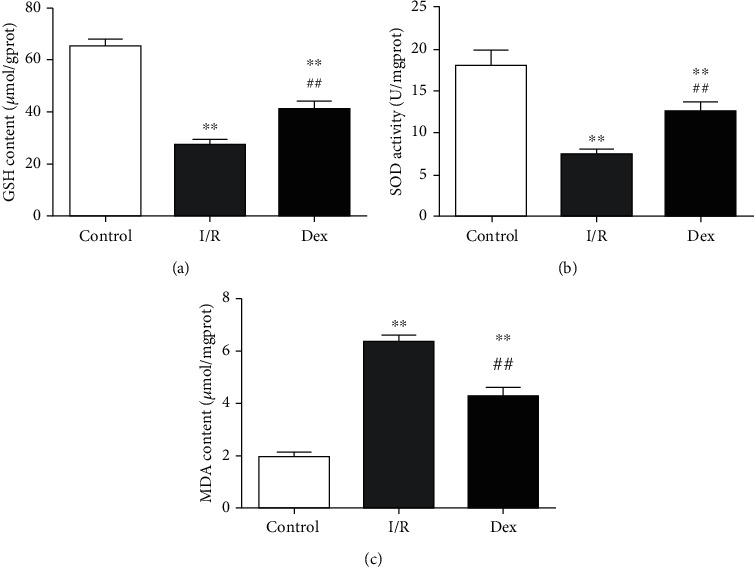
DEX ameliorated I/R-induced oxidative stress. (a) The content of glutathione (GSH) in rat heart tissue. (b) Activities of superoxide dismutase (SOD). (c) The content of malondialdehyde (MDA). Data are shown as mean ± SD (*n* = 6/group). ^∗^*P* < 0.05, ^∗∗^*P* < 0.01 vs. control group. ^#^*P* < 0.05, ^##^*P* < 0.01 vs. I/R group.

To investigate the effect of Dex treatment on I/R-induced oxidative stress, the changes of three critical indicators of oxidative stress were measured, including the content of MDA, GSH, and activity of SOD. As shown in Figure 3, the level of GSH and activity of SOD were strikingly reduced with I/R treatment (GSH 27.50 ± 4.63 *μ*mol/g, SOD 7.48 ± 1.38 U/mg vs. control GSH 65.33 ± 5.92 *μ*mol/g, SOD 18.07 ± 4.54 U/mg, *P* < 0.01, respectively). In contrast, MDA was elevated markedly after I/R (6.38 ± 0.56 *μ*mol/mg vs. 1.94 ± 0.48 *μ*mol/mg in the control group, *P* < 0.01). However, Dex treatment enhanced GSH level (41.17 ± 7.11 *μ*mol/g) and SOD activity (12.54 ± 2.87 U/mg) (vs. I/R, *P* < 0.01, respectively) and significantly decreased MDA level (4.28 ± 0.79 *μ*mol/mg) in the ischemic myocardium (vs. I/R, *P* < 0.01).

### 3.4. Changes in Trx1 and Akt Phosphorylation by Dex in Myocardial I/R Injury

**Figure 4:**
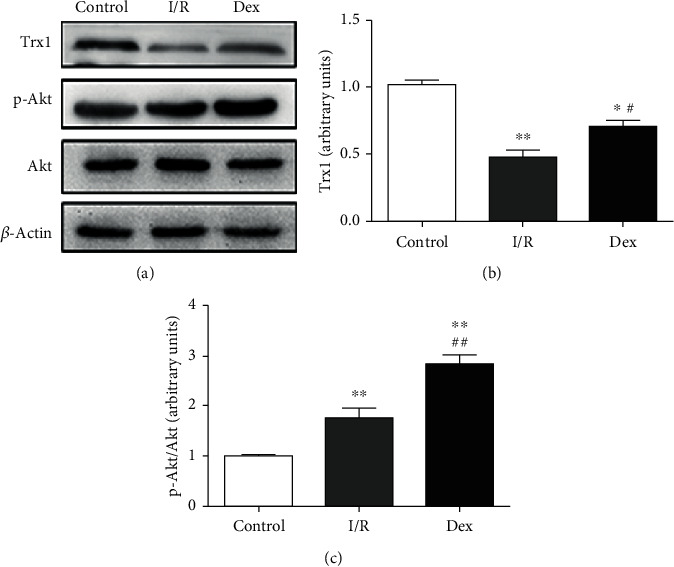
Effects of DEX on expressions of Trx1 and Akt phosphorylation in myocardial I/R rats. (a) Representative Western blots showing the expression of Trx1 and Akt phosphorylation. (b) Quantification of Trx1 expression. (c) Quantitation of the p-Akt/Akt ratio. Data are shown as mean ± SD (*n* = 6/group). ^∗^*P* < 0.05, ^∗∗^*P* < 0.01 vs. control group. ^#^*P* < 0.05, ^##^*P* < 0.01 vs. I/R group.

To further clarify the underlying mechanism, we investigated the role of Dex in Trx1 and Akt phosphorylation in myocardial I/R. The result of Western blotting showed that after I/R injury, Trx1 expression decreased significantly by 0.54-fold compared with the control (*P* < 0.01, Figure 4(b)), while Akt phosphorylation was increased by 1.77-fold (*P* < 0.01, Figure 4(c)). In contrast, Dex treatment increased the expression of Trx1 in the ischemic myocardium by 1.47-fold compared with the I/R group (*P* < 0.05, Figure 4(b)). Meanwhile, Dex treatment also remarkably enhanced the p-Akt/Akt ratio compared to I/R alone (1.60-fold, *P* < 0.01, Figure 4(c)).

### 3.5. PX-12 Abolished the DEX-Induced Cardioprotective Effect on the OGD/R-Treated Myocardium

**Figure 5:**
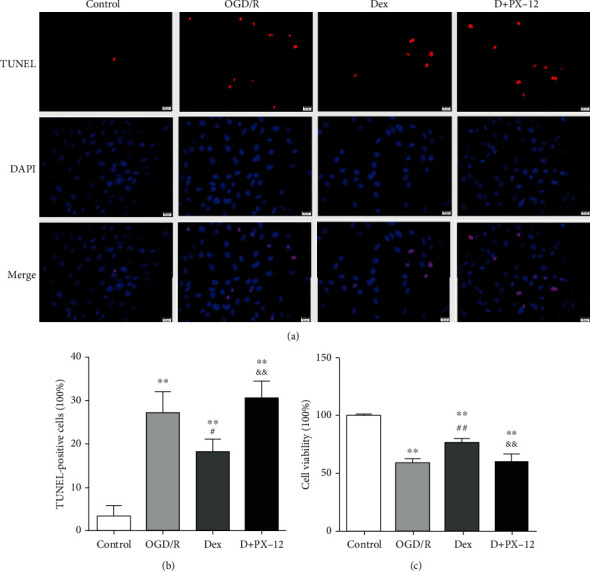
PX-12 blunted the Dex-induced antiapoptotic effect against OGD/R injury in H9c2 cells. H9C2 cells were subjected to OGD/R with or without Dex treatment (1 *μ*mol/L) or pretreatment with Trx1 (5 *μ*mol/L) for 30 min. H9C2 cells were divided into four groups: control, OGD/R, OGD/R+Dex (Dex), and OGD/R+Dex+PX-12 (D+PX-12). (a) Representative images of TUNEL staining (400x). (b) Percentage of TUNEL-positive nuclei. (c) Cellular viability is presented by dividing the optical density of samples by that of the control group. Data are shown as mean ± SD (*n* = 6/group). ^∗^*P* < 0.05, ^∗∗^*P* < 0.01 vs. control group. ^#^*P* < 0.05, ^##^*P* < 0.01 vs. OGD/R group. ^&^*P* < 0.05, ^&&^*P* < 0.01 vs. Dex group.

To further confirm the protective mechanisms of Dex in myocardial I/R injury, we employed PX-12 (a specific Trx1 inhibitor) in our vitro experiment using H9c2 cardiomyocytes treated with OGD/R. OGD/R induced 27.33 ± 4.74% of cell death compared to normoxic conditions (3.37 ± 2.40%) and reduced cell viability to 59.22 ± 3.55% (*P* < 0.01, respectively, Figures 5(b) and 5(c)). In line with previous results, Dex protected against cell death induced by OGD/R (18.14 ± 2.94%); meanwhile, Dex also increased cell viability (76.91 ± 3.06%) as compared with OGD/R-treated cells (*P* < 0.05 and *P* < 0.01, respectively, Figures 5(b) and 5(c)). However, these protective effects were abolished by PX-12 administration, as shown by decreased cell viability (59.90 ± 6.90%) and increased cellular apoptosis (30.68 ± 3.77%) (vs. Dex, *P* < 0.01, respectively, Figures 5(b) and 5(c)).

### 3.6. PX-12 Reversed Dex-Mediated Antioxidation in the OGD/R-Treated Myocardium

**Figure 6:**
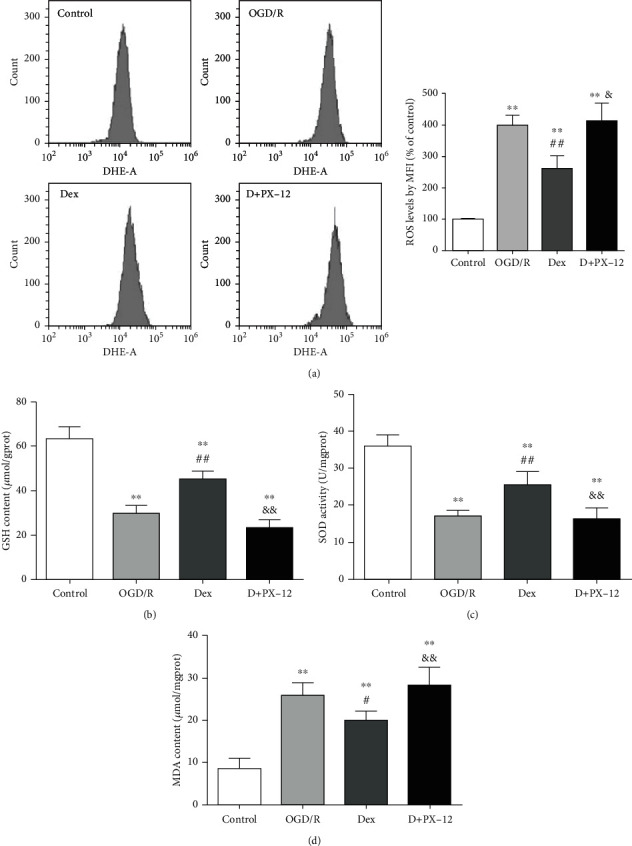
PX-12 inhibited Dex-induced suppression on oxidative damage in OGD/R-treated H9c2 cells. H9C2 cells were divided into four groups: control, OGD/R, Dex, and D+PX-12. (a) ROS levels were determined by flow cytometry. The mean fluorescence intensity (MFI) of 2′,7′-dichlorodihydroflurescein (DCF) was used to reflect cellular ROS levels. (b) The content of GSH in H9C2 cells. (c) Activities of SOD. (d) The content of MDA. Data are shown as mean ± SD (*n* = 6/group). ^∗^*P* < 0.05, ^∗∗^*P* < 0.01 vs. control group. ^#^*P* < 0.05, ^##^*P* < 0.01 vs. OGD/R group. ^&^*P* < 0.05, ^&&^*P* < 0.01 vs. Dex group.

As shown in Figure 6, OGD/R injury caused ROS accumulation by 3.98-fold compared with control H9c2 cardiomyocytes, with increased MDA formation (26.11 ± 2.77 *μ*mol/mg), decreased GSH level (29.81 ± 3.47 *μ*mol/g), and SOD activity (16.93 ± 1.70 U/mg) (vs. control MDA 8.71 ± 2.32 *μ*mol/mg, GSH 63.37 ± 5.21 *μ*mol/g, and SOD 36.00 ± 3.07 U/mg, *P* < 0.01, respectively). Dex significantly reduced the ROS level by 1.52-fold and decreased MDA content (20.06 ± 2.15 *μ*mol/mg), as compared with the OGD/R group (*P* < 0.01, respectively; MDA *P* < 0.05, Figures 6(a)–6(d)); meanwhile, Dex enhanced GSH level (45.26 ± 3.31 *μ*mol/g) and SOD activity (25.97 ± 3.46 U/mg) (vs. OGD/R, *P* < 0.01, respectively, Figures 6(a)–6(d)). However, the inhibition of Trx1 displayed an opposite tendency to the above indicators, as shown by increased ROS level (1.58-fold, vs. Dex, *P* < 0.05), with enhanced MDA formation (28.41 ± 4.12 *μ*mol/mg), reduced level of GSH (23.36 ± 3.62 *μ*mol/g), and SOD activity (16.36 ± 2.92 U/mg) (vs. Dex, *P* < 0.01, respectively, Figures 6(a)–6(d)).

### 3.7. PX-12 Inhibited Myocardial Trx1/Akt Signaling in the OGD/R-Treated Myocardium

**Figure 7:**
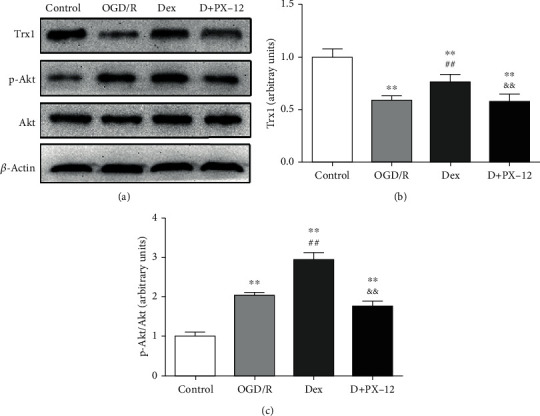
PX-12 reduced expressions of Trx1 and Akt phosphorylation mediated by Dex in OGD/R-treated H9C2 cells. H9C2 cells were divided into four groups: control, OGD/R, Dex, and D+PX-12. (a) Representative Western blots showing the expression of Trx1 and Akt phosphorylation. (b) Quantification of Trx1 expression. (c) Quantitation of the p-Akt/Akt ratio. Data are shown as mean ± SD (*n* = 6/group). ^∗^*P* < 0.05, ^∗∗^*P* < 0.01 vs. control group. ^#^*P* < 0.05, ^##^*P* < 0.01 vs. OGD/R group. ^&^*P* < 0.05, ^&&^*P* < 0.01 vs. Dex group.

Finally, we investigated Trx1/Akt signaling in the OGD/R-treated myocardium. As shown in Figure 7, in agreement with previous results, we found that Dex significantly increased the p-Akt/Akt ratio (1.45-fold) and Trx1 level (1.31-fold) as compared with OGD/R alone (*P* < 0.01, respectively). Nevertheless, these effects were reversed by PX-12 (*P* < 0.01, respectively, Figures 7(b) and 7(c)). Taken together, these results all indicated that Dex protects myocardial I/R injury by ameliorating oxidative stress and cell apoptosis through the Trx1-dependent Akt pathway.

## 4. Discussion

In the present study, we successfully constructed I/R-induced myocardial injury in isolated rat hearts and OGD/R-induced injury in H9c2 cardiomyocytes. The current results have shown that Dex pretreatment alleviated I/R-induced myocardial injury in isolated rat hearts by significantly improving hemodynamic parameters including LVEDP, LVSP, +dP/dtmax, and -dP/dtmin, with limited infarct size and less cellular apoptosis. In vitro, it was also observed that Dex pretreatment reduced OGD/R-induced H9c2 cardiomyocyte apoptosis. Furthermore, our results revealed that DEX pretreatment significantly reduced oxidative stress and myocardial apoptosis by activating the Trx1/Akt pathway in both models. However, these protective effects of Dex were abolished by blocking Trx1. We demonstrated for the first time that Dex could protect the heart from I/R injury through a Trx1-dependent Akt pathway.

Uncontrolled ROS production termed oxidative stress is a major pathogenic factor of I/R injury, resulting in ultrastructural changes of cellular lipids, proteins, enzymes, and DNA via numerous mechanisms [15, 16]. Oxidative stress in I/R injury may lead to an increasing amount of indicative molecular markers of oxidative stress such as MDA [17] and the exhaustion of antioxidants including Trx1, which is an essential cellular antioxidant known to date and fulfills a pivotal role in cardiomyocyte cellular defense of reducing ROS damage [8, 9]. In line with previous studies, we also evidenced a significantly increased oxidative stress after I/R in isolated hearts and H9c2 cardiomyocytes, with a reduction in Trx1 levels, reflected by rapid consumption of GSH and SOD. While Dex treatment remarkably increased the antioxidative enzymes, GSH content and SOD activity decreased MDA and cellular ROS in H9c2 cells, indicating a reduction of oxidative stress in the process of I/R injury. Interestingly, we found that the level of Trx1 was markedly preserved by Dex pretreatment, concomitant with an increase in GSH content and SOD activity and decreased MDA formation. However, this antioxidation effect of Dex was lost when PX-12 inhibited Trx1 in the cellular OGD/R model. These results suggested that Dex could work as a potent antioxidant to relieve the oxidative stress in myocardial I/R injury by rescuing the level of Trx1.

Several studies demonstrate that Akt signaling is involved in the cardioprotective effects conferred by Dex in I/R injury [11, 12, 18]. The Akt signaling pathway plays an important role in regulating cell apoptosis and proliferation by phosphorylating its downstream FOXO1 and mTOR signaling pathway and regulating the activity of the Bcl2/caspase family [19], exhibiting a vital protective effect against myocardial I/R injury [20, 21]. In line with other studies, we also found that Dex treatment enhanced the p-Akt/Akt ratio remarkably with less cell death. Interestingly, it is reported that Trx1 is capable of indirectly phosphorylating Akt by inhibiting PTEN in several pathologies such as myocardial remodeling and cancer [22]. Furthermore, Perez et al. further reported that Trx1 could potentially mediate cardioprotection afforded by both ischemic preconditioning and postconditioning, possibly through an interaction with Akt [23, 24]. Thus, it is interesting to investigate whether increased AKT phosphorylation is related to Trx1 preservation in Dex-induced cardioprotection against I/R injury. In the present study, we found that increased Akt phosphorylation induced by Dex in the cellular OGD/R model was abolished by coadministration with PX-12, leading to more cell apoptosis. This result is in accordance with the notion that Trx1 not only maintains the intracellular redox balance by regenerating active peroxiredoxin but also acts as a signaling molecule to transduce signals to other effectors [8]. Moreover, these results indicated that the activation of Akt by Dex treatment was at least partly through the upregulation of Trx1 in myocardial I/R injury.

Dex is a highly selective *α*2-AR agonist that is widely used in clinical anesthesia and intensive care units. Several clinical studies showed that dexmedetomidine could improve the clinical outcome of patients undergoing cardiac surgery with cardiopulmonary bypass (CPB). The application of dexmedetomidine in cardiac surgery could reduce patients' risks of abnormal hemodynamics. It also benefited patients with cardiac disease undergoing noncardiac surgeries and resulted in less postoperation complications [25, 26]. The results indicated that Dex exhibited a favorable ability of organ protection against myocardial I/R injury. The protection of Dex against I/R injury may be ascribed to its effect on relieving oxidative stress and reducing cellular apoptosis [5, 15, 16]. However, the underlying mechanism is still yet fully understood. As a result, the elucidation of intricate mechanisms for Dex's protection capability is of great clinical significance. Here, we present a novel signaling mechanism that Dex protects myocardial I/R injury by ameliorating oxidative stress and cell apoptosis through the Trx1-dependent Akt pathway, which provides further evidence for Dex's protective effect against myocardial I/R injury. Consequently, Dex is especially indicated to patients with a high risk of myocardial I/R injury or patients undergoing myocardial I/R injury.

While we have proven Dex's myocardial protective effect and identified the Trx1/Akt signaling pathway's possible involvement in this study, several major limitations should be noted. First, this study's findings were obtained from a cellular and an isolated heart I/R model, which are needed to be proven in further in vivo experiments. Second, although we have found that Dex could preserve the level of Trx1 and enhance the activation of Akt in myocardial I/R injury, the underlying interaction mechanism between Dex and Trx1 is still unknown and should be further investigated.

In conclusion, our work revealed that DEX, an *α*2 receptor agonist, protects against myocardial I/R. Specifically, it maintains the capability to improve cardiac function in I/R injury, relieve oxidative stress, and reduce cell apoptosis. Furthermore, the protective effects of DEX in I/R injury are achieved partly through the Trx1-dependent Akt pathway.

## Data Availability

The data used to support the findings of this study are included in the article.
